# Lifetime risk of rheumatoid arthritis-associated interstitial lung disease in *MUC5B* mutation carriers

**DOI:** 10.1136/annrheumdis-2021-220698

**Published:** 2021-08-03

**Authors:** Antti Palomäki, FinnGen Rheumatology Clinical Expert Group, Aarno Palotie, Jukka Koskela, Kari K Eklund, Matti Pirinen, FinnGen, Samuli Ripatti, Tarja Laitinen, Nina Mars

**Affiliations:** 1 Centre for Rheumatology and Clinical Immunology, and Department of Medicine, Turku University Hospital and University of Turku, Turku, Finland; 2 Institute for Molecular Medicine Finland (FIMM), Helsinki Institute of Life Science, University of Helsinki, Helsinki, Finland; 3 Analytic and Translational Genetics Unit, Department of Medicine, Massachusetts General Hospital, Boston, Massachusetts, USA; 4 Stanley Center for Psychiatric Research, Broad Institute of MIT and Harvard, Cambridge, Massachusetts, USA; 5 Department of Rheumatology, University of Helsinki and Helsinki University Hospital, Helsinki, Finland; 6 Orton Orthopaedic Hospital, Helsinki, Finland; 7 Department of Public Health, University of Helsinki, Helsinki, Finland; 8 Department of Mathematics and Statistics, University of Helsinki, Helsinki, Finland; 9 Broad Institute of MIT and Harvard, Cambridge, Massachusetts, USA; 10 Administration Center, Tampere University Hospital, Tampere, Finland

**Keywords:** rheumatoid arthritis, pulmonary fibrosis, polymorphism, genetic

## Abstract

**Objectives:**

To estimate lifetime risk of developing rheumatoid arthritis-associated interstitial lung disease (RA-ILD) with respect to the strongest known risk factor for pulmonary fibrosis, a *MUC5B* promoter variant.

**Methods:**

FinnGen is a collection of epidemiological cohorts and hospital biobank samples, integrating genetic data with up to 50 years of follow-up within nationwide registries in Finland. Patients with RA and ILD were identified from the Finnish national hospital discharge, medication reimbursement and cause-of-death registries. We estimated lifetime risks of ILD by age 80 with respect to the common variant rs35705950, a *MUC5B* promoter variant.

**Results:**

Out of 293 972 individuals, 1965 (0.7%) developed ILD by age 80. Among all individuals in the dataset, *MUC5B* increased the risk of ILD with a HR of 2.44 (95% CI: 2.22 to 2.68). Out of 6869 patients diagnosed with RA, 247 (3.6%) developed ILD. In patients with RA, *MUC5B* was a strong risk factor of ILD with a HR similar to the full dataset (HR: 2.27, 95% CI: 1.75 to 2.95). In patients with RA, lifetime risks of ILD were 16.8% (95% CI: 13.1% to 20.2%) for *MUC5B* carriers and 6.1% (95% CI: 5.0% to 7.2%) for *MUC5B* non-carriers. The difference between risks started to emerge at age 65, with a higher risk among men.

**Conclusion:**

Our findings provide estimates of lifetime risk of RA-ILD based on *MUC5B* mutation carrier status, demonstrating the potential of genomics for risk stratification of RA-ILD.

**Key messages**

**What is already known about this subject?**

- Interstitial lung disease (ILD) is one of the most common extra-articular complications of rheumatoid arthritis (RA). The *MUC5B* promoter variant rs35705950 is an important genetic risk factor for ILD, and case–control studies have identified it to be a risk factor also for RA-ILD.

**What does this study add?**

- By integrating large-scale genotype data with clinical data from nationwide healthcare registries, we show that in patients with RA, *MUC5B* variation is strongly associated with a lifetime risk of RA-ILD.

**How might this impact on clinical practice or future developments?**

- This study highlights the importance of genetic predisposition on the development of RA-ILD. Further studies are needed to investigate the impact of *MUC5B* on outcomes of RA-ILD.

## Introduction

Interstitial lung disease (ILD) is one of the most common extra-articular manifestations of rheumatoid arthritis (RA).^1^ The cumulative risk of developing clinical ILD during the RA disease course has varied in different studies, ranging from 5.0% to 7.7% in long-term follow-up studies of RA cohorts^1–3^ to up to 10% in a study using death records.^4^ Even higher estimates for subclinical radiographic findings consistent with ILD have been observed in patients with RA, ranging from 19% to 33%.^5–7^ Although the RA-ILD course can vary, the disease is associated with significantly increased mortality compared with patients with RA without ILD.^3 4 8^

Clinical risk factors for RA-ILD include older age, male gender, tobacco smoking, high levels of anticitrullinated protein antibodies and disease activity.^2 9^ The strongest known genetic risk factor for idiopathic pulmonary fibrosis (IPF) is the common variant rs35705950, a promoter variant near the *MUC5B* gene.^10^ A recent case–control study has demonstrated that the *MUC5B* promoter variation is associated with an increased risk of ILD among patients with RA.^11^ The aim of this study was to evaluate the lifetime risk of ILD in patients with RA, comparing the risk to the population, and estimate how the *MUC5B* promoter variant modifies these risks in the real-world setting.

## Methods

FinnGen is a collection of prospective epidemiological and disease-based cohorts, and hospital biobank samples. The unique personal identification number links the genotypes to multiple nationwide registries, and cases were identified through the national hospital discharge registry (starting from 1968) including both inpatient and outpatient data, the national death registry (1969–) and the medication reimbursement registry (1964–).

RA was defined as patients having medication reimbursement for inflammatory rheumatic diseases (code 202), with an additional requirement of two contacts with the International Classification of Diseases, Tenth Revision (ICD-10) codes beginning with M05 (seropositive RA) or M06 (seronegative RA). In our recent validation study of RA diagnoses in Finnish biobank patients (unpublished), this combination resulted in a positive predictive value of 0.87 compared with chart review. Negative predictive value for any RA diagnosis was 1.0. Those without RA who had other inflammatory rheumatic diseases or inflammatory bowel disease were excluded.

ILD cases were identified with J84, M05.1/J99.0 (ICD-10), 515, 516 (ICD-9) or 484.99 or 517.01 (ICD-8) with following criteria: (1) the first and only record in the death registry or (2) after the initial diagnosis, a second contact (or death due to ILD) was required within 5 years, that is, we excluded individuals with no further healthcare contacts with ILD within 5 years. No exclusions were made based on temporality of RA and ILD. For both RA and ILD, age at onset was defined as age at first registered diagnosis.

For *MUC5B* (mucin 5B, oligomeric mucus/gel-forming), we studied carriers of the minor allele for the promoter variant rs35705950 (G>T) with minor allele frequency 0.1 (no enrichment compared with non-Finnish Europeans^12^) and mean INFO 0.948 indicating high imputation quality. Individuals homozygous for the variant were analysed jointly with the heterozygotes.

Start of follow-up was set at birth, with follow-up ending at the first record of the endpoint of interest, death, or at the end of follow-up on 31 December 2019, whichever came first. Using the Cox proportional hazards model, we estimated adjusted HRs and 95% CIs (CI). With age as time scale, all regression models were stratified by sex, adjusted for 10 principal components of ancestry, FinnGen genotyping array and cohort. We report cumulative incidences with 95% CIs by age 80. We used R V.3.6.3. Detailed information on genotyping, disease definitions and analyses are provided in online supplemental methods.

10.1136/annrheumdis-2021-220698.supp1 Supplementary data

### Patient and public involvement

This study was carried out without direct patient and public involvement.

## Results

Among 293 972 individuals (mean age at the end of follow-up: 59.8, SD: 17.3, 56.4% women), we identified 1965 patients (1172 men, 793 women) diagnosed with ILD by end of follow-up. Out of 6869 patients with RA (mean age at onset: 49.4, SD: 14.9, 71.1% women), 247 (3.6%) had been diagnosed with ILD. Out of these 247 individuals, 20 (8.1%) had been diagnosed with ILD >1 year before the earliest record of RA, 36 (14.6%) within a year prior to or after the earliest record of RA and 191 (77.3%) >1 year after. Out of patients without RA, 19.3% were *MUC5B* carriers, and out of patients with RA, 20.9%. Among all individuals in the dataset, the *MUC5B* promoter variant rs35705950 was associated with ILD with a HR of 2.44 (2.22–2.68, p=3.87×10^−77^), and among patients with RA, with a HR of 2.27 (1.75–2.95, p=8.15×10^−10^). In a formal test for interaction by introducing an interaction term in the regression model, we found no evidence of an interaction between *MUC5B* and RA (p=0.16). These interaction tests indicate that the effect of *MUC5B* is similar in the population and in patients with RA.

**Figure 1:**
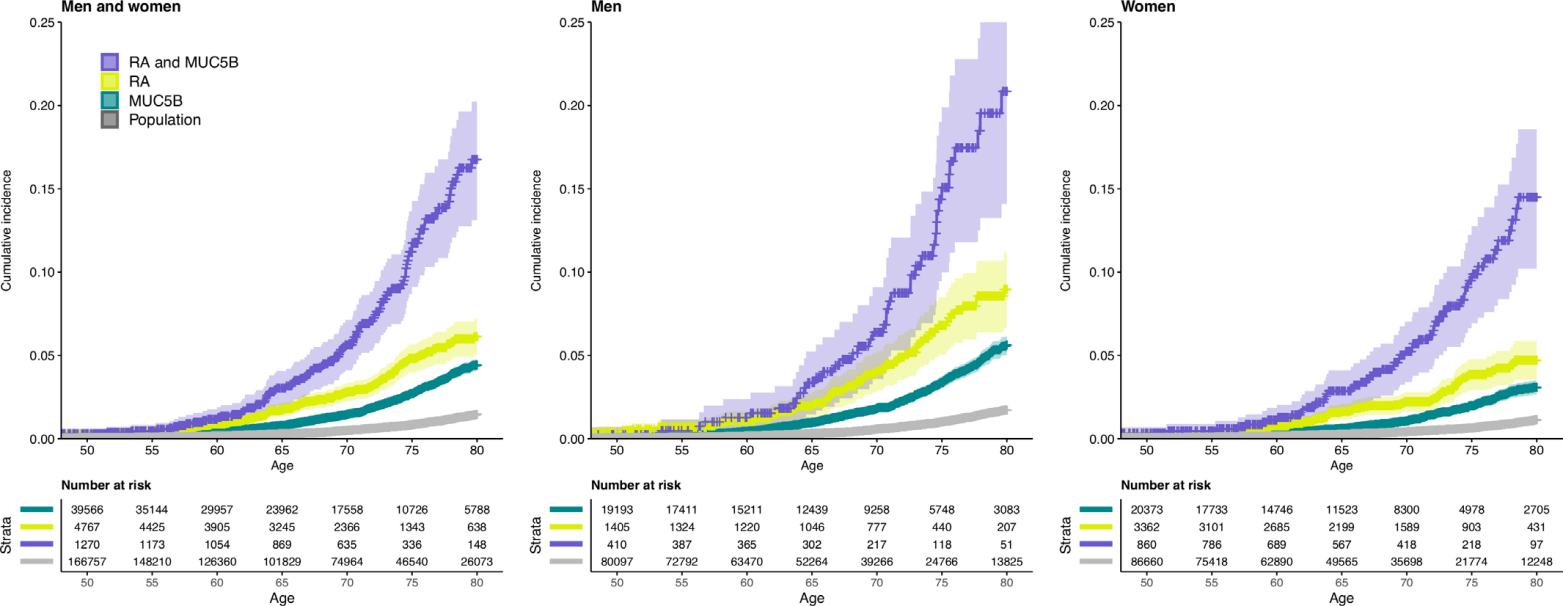
Lifetime risk of interstitial lung disease in the population for *MUC5B* carriers and non-carriers with respect to diagnosis of RA. The risks are shown for men and women both combined and individually. *MUC5B*=carriers of the minor allele for the promoter variant rs35705950. Sample size: 293 972 (128 233 men and 165 739 women). RA, rheumatoid arthritis.

Next, we quantified the lifetime risk of ILD for four groups: (1) *MUC5B* non-carriers in the population, (2) *MUC5B* carriers in the population, (3) *MUC5B* non-carriers with RA and (4) *MUC5B* carriers with RA (figure 1, table 1). The corresponding lifetime risks were (1) 1.5% (95% CI: 1.3% to 1.6%), (2) 4.4% (95% CI: 4.1%–4.8%), 3) 6.1% (95% CI: 5.0%–7.2%) and (4) 16.8% (95% CI: 13.1%–20.2%). In sex-specific analyses, the lifetime risk was 20.9% (95% CI: 14.1%–27.1%) in men with RA who are *MUC5B* carriers, and the corresponding lifetime risk in women was 14.5% (95% CI: 10.2%–18.6%). Accounting for competing risks (non-ILD causes of death) yielded marginally lower estimates of lifetime risks, particularly in men (online supplemental table 1).

**Table 1:** Data characteristics, and effect of RA and *MUC5B* on risk of ILD

	Individuals without RA		Individuals with RA	
	Non-carriers of *MUC5B* promoter variant	Carriers of *MUC5B* promoter variant	Non-carriers of *MUC5B* promoter variant	Carriers of *MUC5B* promoter variant
N	231 860	55 243	5431	1438
ILD cases	1007	711	151	96
ILD cases in men/women	600/407	461/250	70/81	41/55
Age at ILD onset, men/women (mean (SD))	66.9 (10.4)/63.0 (13.3)	67.9 (8.3)/65.3 (11.1)	65.6 (9.1)/64.1 (9.2)	68.5 (7.4)/66.9 (8.7)
Risk of ILD in women and men				
Lifetime risk, % (95% CI)	1.5 (1.3–1.6)	4.4 (4.1–4.8)	6.1 (5.0–7.2)	16.8 (13.1–20.2)
HR (95% CI)	Reference	2.49 (2.25–2.75)	4.99 (4.20–5.94)	9.84 (7.96–12.2)
P value	–	1.24×10^−71^	2.67×10^−74^	4.40×10^−99^
Risk of ILD in men				
Lifetime risk, % (95% CI)	1.7 (1.6–1.9)	5.6 (5.1–6.2)	9.0 (6.7–11.2)	20.9 (14.1–27.1)
HR (95% CI)	Reference	2.63 (2.31–2.98)	5.72 (4.46–7.34)	8.23 (5.96–11.4)
P value	–	2.04×10^−50^	6.81×10^−43^	1.56×10^−37^
Risk of ILD in women				
Lifetime risk, % (95% CI)	1.1 (1.0–1.3)	3.1 (2.6–3.5)	4.7 (3.6–5.9)	14.5 (10.2–18.6)
HR (95% CI)	Reference	2.26 (1.92–2.66)	4.49 (3.53–5.70)	11.9 (8.96–15.8)
P value	–	1.46×10^−22^	1.31×10^−34^	7.86×10^−66^

ILD, interstitial lung disease; RA, rheumatoid arthritis.

Lastly, we observed an association between *MUC5B* and risk of RA (HR: 1.10, 1.04–1.17, p=0.0009), with a somewhat larger association in men (HR: 1.17, 1.05–1.30, p=0.005) than in women (HR: 1.08, 1.01–1.16, p=0.04). The effects remained similar when excluding all 1172 men with ILD (HR: 1.13 in men, 1.01–1.26, p=0.03) and all 793 women with ILD (HR: 1.05 in women, 0.98–1.13, p=0.19). This observation was replicated in UK Biobank (1911 RA cases; see online supplemental methods for details) with a HR of 1.15 (1.03–1.28, p=0.01). Meta-analysing the effects from FinnGen and UK Biobank, the HR was 1.11 (1.06–1.17, p=4.07×10^−5^).

## Discussion

In this large observational cohort study, we demonstrate that a combination of RA and *MUC5B* variation confers a 10-fold elevated risk of ILD compared with the population. Every sixth patient with RA carrying the *MUC5B* risk allele was diagnosed with ILD by age 80, and the risk rapidly increased after age 65. A case–control study by Juge and colleagues recently demonstrated enrichment of *MUC5B* carriers in patients with RA-ILD, with supporting evidence from gene expression in lung parenchyma and high-resolution imaging.^11^ Using large-scale biobank data, we now show how this finding translates to lifetime risks and demonstrate the potential of genomics for risk stratification of RA-ILD and early identification of patients.

Prevalence of RA-ILD shows high variability in the literature depending on the population, diagnostic methods and disease definitions used.^13^ Our lifetime risks compare well with previous estimates of clinically significant disease, reported to occur in up to 5%–10% of patients with RA.^2–4^ We show that the effect of *MUC5B* is similar in the population and in patients with RA, but as both *MUC5B* and RA are important risk factors of ILD, patients with RA who are *MUC5B* carriers are at a much higher risk of ILD than *MUC5B* carriers without RA.

The common variant rs35705950 in the *MUC5B* promoter is strongly associated with upregulation of *MUC5B* expression in the lungs, and the general association between the variant and ILD has been widely replicated.^10 11 14^ In addition, evidence from fine-mapping indicates that rs35705950 might be a causal variant: Bayesian fine-mapping analyses of genome-wide association study (GWAS) results can be used for defining variant sets (credible sets), that with high probability contain one or several causal variants. Several sources report rs35705950 as the only variant in the credible sets for the locus in GWASs on ILD and IPF.^15 16^

We were unable to account for some important risk factors, such as smoking and disease activity, and did not consider other common or rare genetic risk factors,^14 17^ all of which are likely to further contribute to the risk. We did not have information about histological or radiological patterns of ILD. The study was limited to individuals of European ancestry, but *MUC5B* may be a relevant risk factor also in other populations^11^, although many have allele frequencies that are much lower.^12^ With a prevalence of 2.3% for RA and 0.7% for ILD, our sample is slightly enriched in cases, which may affect our estimates. Although ILD was identified through healthcare registries, recurring healthcare encounters were required to reduce the proportion of false positives in our study, and the long-term risk of ILD in patients with RA was in line with previous studies.^1–4^ Patients with RA might be exposed to more chest imaging as part of their standard care and due to increased awareness for the risk of ILD particularly during recent years, which could overestimate the risk difference between patients with and without RA. We also observed a modest association between *MUC5B* and RA, which was replicated in UK Biobank. This association was not detected in a previous study with a smaller sample size by Juge and colleagues.^11^ This tentative finding, which was clearer in men, requires further replication with consideration of other important risk factors, such as smoking. As the effects remained similar when excluding all patients with ILD, we propose that the temporal sequence of ILD and RA is unlikely to impact the association.

In conclusion, the *MUC5B* promoter variant is a common risk factor for ILD in patients with RA and confers a significantly elevated lifetime risk of ILD. This study demonstrates the potential of genomics for risk stratification of RA-ILD and highlights the importance of genetic predisposition on the development of RA-ILD. Studies are needed to further investigate the interaction of clinical and genetic risk factors in the development of RA-ILD, and the impact of *MUC5B* on outcomes of RA-ILD.

## Data Availability

The Finnish biobank data can be accessed through the Fingenious® services (https://site.fingenious.fi/en/) managed by FINBB. The remaining data are available within the article, supplemental information or available from the authors upon request.
