# Supplementary material for: Lifetime risk of rheumatoid arthritis-associated interstitial lung disease in MUC5B mutation carriers
Source: Ann Rheum Dis. 2021 Aug 3;80(12):1530–6. doi: 10.1136/annrheumdis-2021-220698 (PMC8600604; doi:10.1136/annrheumdis-2021-220698)
Supplement: Supplementary data [file annrheumdis-2021-220698supp001.pdf]

Supplementary Table 1. Effect of rheumatoid arthritis (RA) and MUC5B on risk of interstitial lung disease (ILD), accounting for competing risks (non-ILD related death).

	**Population**	***MUC5B***	**RA**	**RA and *MUC5B***
Risk of ILD in men				
Lifetime risk, %	1.5 %	4.8 %	8.5 %	19.5 %
HR (95% CI)	Reference	3.20 (2.84-3.62)	6.00 (4.66-7.73)	11.7 (8.52-16.1)
p-value	-	4.13×10^{-79}	7.21×10^{-44}	1.79×10^{-51}
Risk of ILD in women				
Lifetime risk, %	1.1 %	2.9 %	4.6 %	14.1 %
HR (95% CI)	Reference	2.64 (2.25-3.09)	4.54 (3.57-5.77)	12.6 (9.49-16.7)
p-value	-	1.64×10^{-33}	2.68×10^{-35}	8.91×10^{-70}

Supplementary Table 2. Characteristics of the epidemiological and disease-based cohorts, and hospital biobanks (marked with an asterisk) in the FinnGen dataset used in the study.

	Interstitial lung disease			Rheumatoid arthritis				
	**Number of controls**	**Number of cases**	**Prevalence**	**Number of controls**	**Number of cases**	**Prevalence**	**Mean age at recruitment**	**Mean age at the end of follow-up or death**
AURIA BIOBANK*	31371	208	0.66 %	30433	1146	3.63 %	62.8	64.0
BIOBANK OF CENTRAL FINLAND*	7957	54	0.67 %	7547	464	5.79 %	58.6	59.6
BIOBANK OF EASTERN FINLAND*	9798	62	0.63 %	9456	404	4.10 %	62.2	63.1
BLOOD SERVICE BIOBANK	34330	14	0.04 %	34315	29	0.08 %	43.6	44.7
BOREALIS BIOBANK*	8657	96	1.10 %	8372	381	4.35 %	62.6	63.8
HELSINKI BIOBANK*	60819	502	0.82 %	59440	1881	3.07 %	56.9	58.3
TAMPERE BIOBANK*	20147	218	1.07 %	19725	640	3.14 %	56.7	58.1
TERVEYSTALO BIOBANK	6644	11	0.17 %	6498	157	2.36 %	51.0	50.7
THL BIOBANK ATBC	8446	102	1.19 %	8472	76	0.89 %	62.9	77.0
THL BIOBANK BOTNIA	11441	40	0.35 %	11348	133	1.16 %	51.5	68.3
THL BIOBANK COROGENE	4377	69	1.55 %	4346	100	2.25 %	65.8	75.6
THL BIOBANK FINHEALTH 2017	5719	15	0.26 %	5648	86	1.50 %	54.6	57.4
THL BIOBANK FINRISK 1992	4760	32	0.67 %	4685	107	2.23 %	44.2	69.7
THL BIOBANK FINRISK 1997	6799	48	0.70 %	6712	135	1.97 %	48.3	68.9
THL BIOBANK FINRISK 2002	6689	34	0.51 %	6605	118	1.76 %	47.9	64.8
THL BIOBANK FINRISK 2007	4969	27	0.54 %	4896	100	2.00 %	51.2	63.6
THL BIOBANK FINRISK 2012	5093	12	0.24 %	5021	84	1.65 %	51.1	58.8
THL BIOBANK HEALTH 2000	6293	19	0.30 %	6190	122	1.93 %	55.7	72.0
THL BIOBANK HHS	3253	39	1.18 %	3266	26	0.79 %	47.5	79.0
THL BIOBANK MIGRAINE	7700	28	0.36 %	7610	118	1.53 %	44.8	58.6
THL BIOBANK SUPER	8249	16	0.19 %	8222	43	0.52 %	46.9	49.3
THL BIOBANK T1D	9891	28	0.28 %	9701	218	2.20 %	32.7	52.0
THL BIOBANK TWINS	11003	98	0.88 %	10892	209	1.88 %	48.5	68.9
Other	7602	193	2.48 %	7703	92	1.18 %	54.8	58.8

Supplementary Table 3. Diseases excluded from the study. We excluded from all analyses 11,808 individuals without rheumatoid arthritis who had at least one record for any of these diseases.

	Hospital discharge registry			Hospital discharge registry codes to exclude	Cause of death registry			Cause of death registry codes to exclude	Kela* reimbursement codes
	ICD-10	ICD-9	ICD-8	ICD-9	ICD-10	ICD-9	ICD-8	ICD-9	
**Systemic lupus erythematosus**	M32	7100	7431		M32	7100	7431		
**Inflammatory bowel disease**									
Crohn disease	K50	555	5630		K50	555	5630		
Ulcerative colitis	K51	556	5631 or 569	5564A	K51	556	5631 or 569	5564A	
IBD patients in Kela*-register									208 or 209
**Systemic sclerosis**	M34	7101	7340		M34	7101	7340		
**Ankylosing spondylitis**	M45	7200	7124		M45	7200	7124		
**Dermatopolymyositis**	M33				M33				
Juvenile dermatomyositis	M330	7103A			M330	7103A			
Other dermatomyositis	M331	7103B	7160		M331	7103B	7160		
Polymyositis	M332	7104	7161		M332	7104	7161		
Dermatopolymyositis, unspecified	M339				M339				
**Myositis**	M60	7280	732		M60	7280	732		

*KELA = the Social insurance Institute of Finland

Supplementary Methods

Diseases

FinnGen is a collection of prospective epidemiological and disease-based cohorts and hospital biobank samples, aiming for a collection of 500,000 genotype samples from Finnish individuals by 2023. The study uses FinnGen data freeze 7. Further information about the subcohorts included in FinnGen can be found in **Supplementary Table 2**.

Age at RA onset was defined through the first registered diagnosis in the registries (age ≥16 at diagnosis accepted as case). Age at ILD diagnosis was defined through the first registered diagnosis in the registries (at death, if death was the only event; age ≥16 at diagnosis accepted as case, with 8 individuals excluded due to diagnosis under age 16). 630 individuals were excluded due to no further healthcare contacts with ILD within 5 years after the initial diagnosis (individuals with ILD death as first event considered as cases). Competing risks were defined as any non-ILD related cause of death identified from the registry on causes of death. We excluded from all analyses a further 11,808 individuals without RA who had at least one record for any of the following diseases: systemic lupus erythematosus, inflammatory bowel disease, systemic sclerosis, ankylosing spondylitis, dermatopolymyositis, or myositis (**Supplementary Table 3**). The full FinnGen Data Freeze 7 consists of 306,418 adults (age ≥18), and with the exclusions listed above, the final sample size was 293,972.

Genotyping and imputation

The samples are genotyped with Illumina and Affymetrix arrays (Illumina Inc., San Diego, and Thermo Fisher Scientific, Santa Clara, CA, USA). Individuals with ambiguous gender, high genotype missingness (>5%), excess heterozygosity (+-4SD) and non-Finnish ancestry were excluded, as well as all variants with high missingness (>2%), low Hardy–Weinberg equilibrium p-value (<1e-6) and minor allele count (MAC < 3). Array data pre-phasing was carried out with Eagle 2.3.527 with the number of conditioning haplotypes set to 20,000. The genotypes have been imputed with using the SISu v3 population-specific reference panel developed from high-quality data for 3,775 high-coverage (25-30x) whole-genome sequencing in Finns. The detailed genotype imputation workflow can be found at https://dx.doi.org/10.17504/protocols.io.xbgfijw. The dataset uses genome build 38 (hg38). Variant extraction from VCF files with PLINK v2.00a2.3LM.45 using --export A. The HWE p-value for rs35705950 was 0.18.

Replication in UK Biobank

UK Biobank is a prospective cohort study comprising approximately 500,000 individuals from across the United Kingdom, aged between 40 and 69 at recruitment. The cohort contains deep phenotyping, including biological measurements, lifestyle factors, and blood biomarkers. The dataset has been imputed using the merged UK10K and 1000 Genomes (phase 3) reference panels.[1] Details on the cohort, as well as data generation and imputation have been previously described.[2] RA was defined as at least two contacts with ICD-10 codes beginning with M05 (seropositive RA) or M06 (seronegative RA) in the inpatient hospital data (data field 41270; age for earliest visit from data field 41280). The sample size in the UK Biobank analysis was 343,676 (White British individuals unrelated based on KING kinship coefficient <0.0442). We identified 1,911 RA cases (1,368 women), with their mean age at RA onset at 58.5 years (s.d. 8.9 years). This analysis was conducted with the UK Biobank Resource under Application Number 22627.

Statistical analysis

With age as time scale, all regression models were stratified by sex, adjusting for ten principal components of ancestry, as well as genotyping array (FinnGen and UK Biobank), cohort (FinnGen), and batch (UK Biobank). The proportional hazards assumption was evaluated by Schoenfeld residuals and log–log inspection. Meta-analysis was performed by inverse-variance weighting. Competing risk models were performed by fitting the Fine and Gray proportional subdistribution hazards regression model using the R package *cmprsk*. Function *crr()* was used for obtaining sex-specific effect sizes with 95% confidence intervals (CIs) with the covariates inputted as a numerical covariate matrix. The lifetime risks were estimated with *cuminc(),* which estimates the cumulative incidence functions from the output generated by *crr()*. All tests were two-tailed.

Supplementary References

1. Huang, J., *et al*. Improved imputation of low-frequency and rare variants using the UK10K haplotype reference panel. *Nat Commun* **6**, 8111 (2015).
2. Bycroft, C., *et al*. The UK Biobank resource with deep phenotyping and genomic data. *Nature* **562**, 203-209 (2018).

FinnGen Rheumatology Clinical Expert Group

Kari Eklund	Hospital District of Helsinki and Uusimaa, Helsinki, Finland
Antti Palomäki	Hospital District of Southwest Finland, Turku, Finland
Pia Isomaki	Pirkanmaa Hospital District, Tampere, Finland
Laura Pirilä	Hospital District of Southwest Finland, Turku, Finland
Oili Kaipiainen-Seppänen	Northern Savo Hospital District, Kuopio, Finland
Johanna Huhtakangas	Northern Savo Hospital District, Kuopio, Finland
Ali Abbasi	Abbvie, Chicago, IL, United States
Jeffrey Waring	Abbvie, Chicago, IL, United States
Fedik Rahimov	Abbvie, Chicago, IL, United States
Apinya Lertratanakul	Abbvie, Chicago, IL, United States
Nizar Smaoui	Abbvie, Chicago, IL, United States
Anne Lehtonen	Abbvie, Chicago, IL, United States
Adam Platt	Astra Zeneca, Cambridge, United Kingdom
David Close	Astra Zeneca, Cambridge, United Kingdom
Marla Hochfeld	Celgene, Summit, NJ, United States/Bristol Myers Squibb, New York, NY, United States
Natalie Bowers	Genentech, San Francisco, CA, United States
Sarah Pendergrass	Genentech, San Francisco, CA, United States
Onuralp Soylemez	Merck, Kenilworth, NJ, United States
Kirsi Kalpala	Pfizer, New York, NY, United States
Nan Bing	Pfizer, New York, NY, United States
Xinli Hu	Pfizer, New York, NY, United States
Kirsi Auro	GlaxoSmithKline, Brentford, United Kingdom
Dawn Waterworth	Janssen Biotech, Beerse, Belgium
Andrea Ganna	Institute for Molecular Medicine Finland, HiLIFE, Helsinki, Finland, Finland
Anne Kerola	University of Helsinki
Hanna-Kaisa Heikkilä	Pirkanmaa Hospital District, Tampere, Finland
Javier Gracia Tabuenca	University of Tampere
Johanna Hiltunen	Hospital District of Helsinki and Uusimaa, Helsinki, Finland
Johanna Paltta	Hospital District of Southwest Finland, Turku, Finland
Juha Sinisalo	Hospital District of Helsinki and Uusimaa, Helsinki, Finland
Jukka Koskela	Institute for Molecular Medicine Finland, HiLIFE, Helsinki, Finland, Finland
Nina Mars	nstitute for Molecular Medicine Finland, HiLIFE, Helsinki, Finland, Finland
Tarja Laitinen	University of Tampere
Tuulikki Sokka-Isler	Jyväskylä Central Hospital
Vincent Llorens	Institute for Molecular Medicine Finland, HiLIFE, Helsinki, Finland, Finland

FinnGen

Steering Committee

Aarno Palotie Institute for Molecular Medicine Finland, HiLIFE, University of Helsinki, Finland
Mark Daly Institute for Molecular Medicine Finland, HiLIFE, University of Helsinki, Finland

Pharmaceutical companies

Bridget Riley-Gills Abbvie, Chicago, IL, United States
Howard Jacob Abbvie, Chicago, IL, United States
Dirk Paul Astra Zeneca, Cambridge, United Kingdom
Heiko Runz Biogen, Cambridge, MA, United States
Sally John Biogen, Cambridge, MA, United States
Robert Plenge Celgene, Summit, NJ, United States/Bristol Myers Squibb, New York, NY, United States
Mark McCarthy Genentech, San Francisco, CA, United States
Julie Hunkapiller Genentech, San Francisco, CA, United States
Meg Ehm GlaxoSmithKline, Brentford, United Kingdom
Kirsi Auro GlaxoSmithKline, Brentford, United Kingdom
Caroline Fox Merck, Kenilworth, NJ, United States
Anders Mälarstig Pfizer, New York, NY, United States
Katherine Klinger Sanofi, Paris, France
Deepak Raipal Sanofi, Paris, France
Tim Behrens Maze Therapeutics, San Francisco, CA, United States
Robert Yang Janssen Biotech, Beerse, Belgium
Richard Siegel Novartis, Basel, Switzerland

University of Helsinki & Biobanks

Tomi Mäkelä HiLIFE, University of Helsinki, Finland, Finland
Jaakko Kaprio Institute for Molecular Medicine Finland, HiLIFE, Helsinki, Finland, Finland
Petri Virolainen Auria Biobank / University of Turku / Hospital District of Southwest Finland, Turku, Finland
Antti Hakanen Auria Biobank / University of Turku / Hospital District of Southwest Finland, Turku, Finland
Terhi Kilpi THL Biobank / The National Institute of Health and Welfare Helsinki, Finland
Markus Perola THL Biobank / The National Institute of Health and Welfare Helsinki, Finland
Jukka Partanen Finnish Red Cross Blood Service / Finnish Hematology Registry and Clinical Biobank, Helsinki, Finland
Anne Pitkäranta Helsinki Biobank / Helsinki University and Hospital District of Helsinki and Uusimaa, Helsinki
Juhani Junttila Northern Finland Biobank Borealis / University of Oulu / Northern Ostrobothnia Hospital District, Oulu, Finland
Raisa Serpi Northern Finland Biobank Borealis / University of Oulu / Northern Ostrobothnia Hospital District, Oulu, Finland
Tarja Laitinen Finnish Clinical Biobank Tampere / University of Tampere / Pirkanmaa Hospital District, Tampere, Finland

Johanna Mäkelä Finnish Clinical Biobank Tampere / University of Tampere / Pirkanmaa Hospital District, Tampere, Finland
Veli-Matti Kosma Biobank of Eastern Finland / University of Eastern Finland / Northern Savo Hospital District, Kuopio, Finland
Urho Kujala Central Finland Biobank / University of Jyväskylä / Central Finland Health Care District, Jyväskylä, Finland

Other Experts/ Non-Voting Members
Outi Tuovila Business Finland, Helsinki, Finland
Raimo Pakkanen Business Finland, Helsinki, Finland

Scientific Committee
Pharmaceutical companies
Jeffrey Waring Abbvie, Chicago, IL, United States
Ali Abbasi Abbvie, Chicago, IL, United States
Mengzhen Liu Abbvie, Chicago, IL, United States
Ioanna Tachmazidou Astra Zeneca, Cambridge, United Kingdom
Chia-Yen Chen Biogen, Cambridge, MA, United States
Heiko Runz Biogen, Cambridge, MA, United States
Shameek Biswas Celgene, Summit, NJ, United States/Bristol Myers Squibb, New York, NY, United States
Julie Hunkapiller Genentech, San Francisco, CA, United States
Meg Ehm GlaxoSmithKline, Brentford, United Kingdom
Neha Raghavan Merck, Kenilworth, NJ, United States
Adriana Huertas-Vazquez Merck, Kenilworth, NJ, United States
Anders Mälarstig Pfizer, New York, NY, United States
Xinli Hu Pfizer, New York, NY, United States
Katherine Klinger Sanofi, Paris, France
Matthias Gossel Sanofi, Paris, France
Robert Graham Maze Therapeutics, San Francisco, CA, United States
Tim Behrens Maze Therapeutics, San Francisco, CA, United States
Beryl Cummings Maze Therapeutics, San Francisco, CA, United States
Wilco Fleuren Janssen Biotech, Beerse, Belgium
Dawn Waterworth Janssen Biotech, Beerse, Belgium
Nicole Renaud Novartis, Basel, Switzerland
Ma´en Obeidat Novartis, Basel, Switzerland

University of Helsinki & Biobanks
Samuli Ripatti Institute for Molecular Medicine Finland, HiLIFE, Helsinki, Finland
Johanna Schleutker Auria Biobank / Univ. of Turku / Hospital District of Southwest Finland, Turku, Finland
Markus Perola THL Biobank / The National Institute of Health and Welfare Helsinki, Finland
Mikko Arvas Finnish Red Cross Blood Service / Finnish Hematology Registry and Clinical Biobank, Helsinki, Finland
Olli Carpén Helsinki Biobank / Helsinki University and Hospital District of Helsinki and Uusimaa, Helsinki

Reetta Hinttala Northern Finland Biobank Borealis / University of Oulu / Northern Ostrobothnia Hospital District, Oulu, Finland
Johannes Kettunen Northern Finland Biobank Borealis / University of Oulu / Northern Ostrobothnia Hospital District, Oulu, Finland
Johanna Mäkelä Finnish Clinical Biobank Tampere / University of Tampere / Pirkanmaa Hospital District, Tampere, Finland
Arto Mannermaa Biobank of Eastern Finland / University of Eastern Finland / Northern Savo Hospital District, Kuopio, Finland
Jari Laukkanen Central Finland Biobank / University of Jyväskylä / Central Finland Health Care District, Jyväskylä, Finland
Urho Kujala Central Finland Biobank / University of Jyväskylä / Central Finland Health Care District, Jyväskylä, Finland

Clinical Groups

Neurology Group

Reetta Kälviäinen Northern Savo Hospital District, Kuopio, Finland
Valtteri Julkunen Northern Savo Hospital District, Kuopio, Finland
Hilkka Soininen Northern Savo Hospital District, Kuopio, Finland
Anne Remes Northern Ostrobothnia Hospital District, Oulu, Finland
Mikko Hiltunen Northern Savo Hospital District, Kuopio, Finland
Jukka Peltola Pirkanmaa Hospital District, Tampere, Finland
Pentti Tienari Hospital District of Helsinki and Uusimaa, Helsinki, Finland
Juha Rinne Hospital District of Southwest Finland, Turku, Finland
Roosa Kallionpää Hospital District of Southwest Finland, Turku, Finland
Ali Abbasi Abbvie, Chicago, IL, United States
Adam Ziemann Abbvie, Chicago, IL, United States
Jeffrey Waring Abbvie, Chicago, IL, United States
Sahar Esmaeeli Abbvie, Chicago, IL, United States
Nizar Smaoui Abbvie, Chicago, IL, United States
Anne Lehtonen Abbvie, Chicago, IL, United States
Susan Eaton Biogen, Cambridge, MA, United States
Heiko Runz Biogen, Cambridge, MA, United States
Sanni Lahdenperä Biogen, Cambridge, MA, United States
Janet van Adelsberg Celgene, Summit, NJ, United States/ Bristol Myers Squibb, New York, NY, United States
Shameek Biswas Celgene, Summit, NJ, United States/ Bristol Myers Squibb, New York, NY, United States
Julie Hunkapiller Genentech, San Francisco, CA, United States
Natalie Bowers Genentech, San Francisco, CA, United States
Edmond Teng Genentech, San Francisco, CA, United States
Sarah Pendergrass Genentech, San Francisco, CA, United States
Onuralp Soylemez Merck, Kenilworth, NJ, United States
Kari Linden Pfizer, New York, NY, United States
Fanli Xu GlaxoSmithKline, Brentford, United Kingdom
David Pulford GlaxoSmithKline, Brentford, United Kingdom
Kirsi Auro GlaxoSmithKline, Brentford, United Kingdom
Laura Addis GlaxoSmithKline, Brentford, United Kingdom

John Eicher GlaxoSmithKline, Brentford, United Kingdom
Minna Raivio Hospital District of Helsinki and Uusimaa, Helsinki, Finland
Sarah Pendergrass Genentech, San Francisco, CA, United States
Beryl Cummings Maze Therapeutics, San Francisco, CA, United States
Juulia Partanen Institute for Molecular Medicine Finland, HiLIFE, University of Helsinki, Finland

Gastroenterology Group
Martti Färkkilä Hospital District of Helsinki and Uusimaa, Helsinki, Finland
Jukka Koskela Hospital District of Helsinki and Uusimaa, Helsinki, Finland
Sampsa Pikkarainen Hospital District of Helsinki and Uusimaa, Helsinki, Finland
Airi Jussila Pirkanmaa Hospital District, Tampere, Finland
Katri Kaukinen Pirkanmaa Hospital District, Tampere, Finland
Timo Blomster Northern Ostrobothnia Hospital District, Oulu, Finland
Mikko Kiviniemi Northern Savo Hospital District, Kuopio, Finland
Markku Voutilainen Hospital District of Southwest Finland, Turku, Finland
Ali Abbasi Abbvie, Chicago, IL, United States
Graham Heap Abbvie, Chicago, IL, United States
Jeffrey Waring Abbvie, Chicago, IL, United States
Nizar Smaoui Abbvie, Chicago, IL, United States
Fedik Rahimov Abbvie, Chicago, IL, United States
Anne Lehtonen Abbvie, Chicago, IL, United States
Keith Usiskin Celgene, Summit, NJ, United States/ Bristol Myers Squibb, New York, NY, United States
Tim Lu Genentech, San Francisco, CA, United States
Natalie Bowers Genentech, San Francisco, CA, United States
Danny Oh Genentech, San Francisco, CA, United States
Sarah Pendergrass Genentech, San Francisco, CA, United States
Kirsi Kalpala Pfizer, New York, NY, United States
Melissa Miller Pfizer, New York, NY, United States
Xinli Hu Pfizer, New York, NY, United States
Linda McCarthy GlaxoSmithKline, Brentford, United Kingdom
Onuralp Soylemez Merck, Kenilworth, NJ, United States
Mark Daly Institute for Molecular Medicine Finland, HiLIFE, University of Helsinki, Finland

Pulmonology Group
Tarja Laitinen Pirkanmaa Hospital District, Tampere, Finland
Margit Pelkonen Northern Savo Hospital District, Kuopio, Finland
Paula Kauppi Hospital District of Helsinki and Uusimaa, Helsinki, Finland
Hannu Kankaanranta Pirkanmaa Hospital District, Tampere, Finland
Terttu Harju Northern Ostrobothnia Hospital District, Oulu, Finland
Riitta Lahesmaa Hospital District of Southwest Finland, Turku, Finland
Nizar Smaoui Abbvie, Chicago, IL, United States
Alex Mackay Astra Zeneca, Cambridge, United Kingdom
Glenda Lassi Astra Zeneca, Cambridge, United Kingdom
Susan Eaton Biogen, Cambridge, MA, United States

Steven Greenberg Celgene, Summit, NJ, United States/ Bristol Myers Squibb, New York, NY, United States
Hubert Chen Genentech, San Francisco, CA, United States
Sarah Pendergrass Genentech, San Francisco, CA, United States
Natalie Bowers Genentech, San Francisco, CA, United States
Joanna Betts GlaxoSmithKline, Brentford, United Kingdom
Soumitra Ghosh GlaxoSmithKline, Brentford, United Kingdom
Kirsi Auro GlaxoSmithKline, Brentford, United Kingdom
Rajashree Mishra GlaxoSmithKline, Brentford, United Kingdom
Sina Rüeger Institute for Molecular Medicine Finland, HiLIFE, University of Helsinki, Finland

Cardiometabolic Diseases Group

Teemu Niiranen The National Institute of Health and Welfare Helsinki, Finland
Felix Vaura The National Institute of Health and Welfare Helsinki, Finland
Veikko Salomaa The National Institute of Health and Welfare Helsinki, Finland
Markus Juonala Hospital District of Southwest Finland, Turku, Finland
Kaj Metsärinne Hospital District of Southwest Finland, Turku, Finland
Mika Kähönen Pirkanmaa Hospital District, Tampere, Finland
Juhani Junttila Northern Ostrobothnia Hospital District, Oulu, Finland
Markku Laakso Northern Savo Hospital District, Kuopio, Finland
Jussi Pihlajamäki Northern Savo Hospital District, Kuopio, Finland
Daniel Gordin Hospital District of Helsinki and Uusimaa, Helsinki, Finland
Juha Sinisalo Hospital District of Helsinki and Uusimaa, Helsinki, Finland
Marja-Riitta Taskinen Hospital District of Helsinki and Uusimaa, Helsinki, Finland
Tiinamaija Tuomi Hospital District of Helsinki and Uusimaa, Helsinki, Finland
Jari Laukkanen Central Finland Health Care District, Jyväskylä, Finland
Benjamin Challis Astra Zeneca, Cambridge, United Kingdom
Dirk Paul Astra Zeneca, Cambridge, United Kingdom
Julie Hunkapiller Genentech, San Francisco, CA, United States
Natalie Bowers Genentech, San Francisco, CA, United States
Sarah Pendergrass Genentech, San Francisco, CA, United States
Onuralp Soylemez Merck, Kenilworth, NJ, United States
Jaakko Parkkinen Pfizer, New York, NY, United States
Melissa Miller Pfizer, New York, NY, United States
Russell Miller Pfizer, New York, NY, United States
Audrey Chu GlaxoSmithKline, Brentford, United Kingdom
Kirsi Auro GlaxoSmithKline, Brentford, United Kingdom
Keith Usiskin Celgene, Summit, NJ, United States/ Bristol Myers Squibb, New York, NY, United States
Amanda Elliott Institute for Molecular Medicine Finland, HiLIFE, University of Helsinki, Finland / Broad Institute, Cambridge, MA, United States
Joel Rämö Institute for Molecular Medicine Finland, HiLIFE, University of Helsinki, Finland
Samuli Ripatti Institute for Molecular Medicine Finland, HiLIFE, University of Helsinki, Finland

Mary Pat Reeve Institute for Molecular Medicine Finland, HiLIFE, University of Helsinki, Finland
Sanni Ruotsalainen Institute for Molecular Medicine Finland, HiLIFE, University of Helsinki, Finland

Oncology Group
Tuomo Meretoja Hospital District of Helsinki and Uusimaa, Helsinki, Finland
Heikki Joensuu Hospital District of Helsinki and Uusimaa, Helsinki, Finland
Olli Carpén Hospital District of Helsinki and Uusimaa, Helsinki, Finland
Lauri Aaltonen Hospital District of Helsinki and Uusimaa, Helsinki, Finland
Johanna Mattson Hospital District of Helsinki and Uusimaa, Helsinki, Finland
Annika Auranen Pirkanmaa Hospital District , Tampere, Finland
Peeter Karihtala Northern Ostrobothnia Hospital District, Oulu, Finland
Saila Kauppila Northern Ostrobothnia Hospital District, Oulu, Finland
Päivi Auvinen Northern Savo Hospital District, Kuopio, Finland
Klaus Elenius Hospital District of Southwest Finland, Turku, Finland
Johanna Schleutker Hospital District of Southwest Finland, Turku, Finland
Relja Popovic Abbvie, Chicago, IL, United States
Jeffrey Waring Abbvie, Chicago, IL, United States
Bridget Riley-Gillis Abbvie, Chicago, IL, United States
Anne Lehtonen Abbvie, Chicago, IL, United States
Jennifer Schutzman Genentech, San Francisco, CA, United States
Julie Hunkapiller Genentech, San Francisco, CA, United States
Natalie Bowers Genentech, San Francisco, CA, United States
Sarah Pendergrass Genentech, San Francisco, CA, United States
Andrey Loboda Merck, Kenilworth, NJ, United States
Aparna Chhibber Merck, Kenilworth, NJ, United States
Heli Lehtonen Pfizer, New York, NY, United States
Stefan McDonough Pfizer, New York, NY, United States
Marika Crohns Sanofi, Paris, France
Sauli Vuoti Sanofi, Paris, France
Diptee Kulkarni GlaxoSmithKline, Brentford, United Kingdom
Kirsi Auro GlaxoSmithKline, Brentford, United Kingdom
Esa Pitkänen Institute for Molecular Medicine Finland, HiLIFE, University of Helsinki, Finland
Nina Mars Institute for Molecular Medicine Finland, HiLIFE, University of Helsinki, Finland
Mark Daly Institute for Molecular Medicine Finland, HiLIFE, University of Helsinki, Finland

Opthalmology Group
Kai Kaarniranta Northern Savo Hospital District, Kuopio, Finland
Joni A Turunen Hospital District of Helsinki and Uusimaa, Helsinki, Finland
Terhi Ollila Hospital District of Helsinki and Uusimaa, Helsinki, Finland
Sanna Seitsonen Hospital District of Helsinki and Uusimaa, Helsinki, Finland
Hannu Uusitalo Pirkanmaa Hospital District, Tampere, Finland
Vesa Aaltonen Hospital District of Southwest Finland, Turku, Finland
Hannele Uusitalo-Järvinen Pirkanmaa Hospital District, Tampere, Finland

Marja Luodonpää Northern Ostrobothnia Hospital District, Oulu, Finland
Nina Hautala Northern Ostrobothnia Hospital District, Oulu, Finland
Mengzhen Liu Abbvie, Chicago, IL, United States
Heiko Runz Biogen, Cambridge, MA, United States
Stephanie Loomis Biogen, Cambridge, MA, United States
Erich Strauss Genentech, San Francisco, CA, United States
Natalie Bowers Genentech, San Francisco, CA, United States
Hao Chen Genentech, San Francisco, CA, United States
Sarah Pendergrass Genentech, San Francisco, CA, United States
Anna Podgornaia Merck, Kenilworth, NJ, United States
Juha Karjalainen Institute for Molecular Medicine Finland, HiLIFE, University of Helsinki, Finland / Broad Institute, Cambridge, MA, United States
Esa Pitkänen Institute for Molecular Medicine Finland, HiLIFE, University of Helsinki, Finland

Dermatology Group
Kaisa Tasanen Northern Ostrobothnia Hospital District, Oulu, Finland
Laura Huilaja Northern Ostrobothnia Hospital District, Oulu, Finland
Katariina Hannula-Jouppi Hospital District of Helsinki and Uusimaa, Helsinki, Finland
Teea Salmi Pirkanmaa Hospital District, Tampere, Finland
Sirkku Peltonen Hospital District of Southwest Finland, Turku, Finland
Leena Koulu Hospital District of Southwest Finland, Turku, Finland
Kirsi Kalpala Pfizer, New York, NY, United States
Ying Wu Pfizer, New York, NY, United States
David Choy Genentech, San Francisco, CA, United States
Sarah Pendergrass Genentech, San Francisco, CA, United States
Nizar Smaoui Abbvie, Chicago, IL, United States
Fedik Rahimov Abbvie, Chicago, IL, United States
Anne Lehtonen Abbvie, Chicago, IL, United States
Dawn Waterworth Janssen Biotech, Beerse, Belgium

Odontology Group
Pirkko Pussinen Hospital District of Helsinki and Uusimaa, Helsinki, Finland
Aino Salminen Hospital District of Helsinki and Uusimaa, Helsinki, Finland
Tuula Salo Hospital District of Helsinki and Uusimaa, Helsinki, Finland
David Rice Hospital District of Helsinki and Uusimaa, Helsinki, Finland
Pekka Nieminen Hospital District of Helsinki and Uusimaa, Helsinki, Finland
Ulla Palotie Hospital District of Helsinki and Uusimaa, Helsinki, Finland
Juha Sinisalo Hospital District of Helsinki and Uusimaa, Helsinki, Finland
Maria Siponen Northern Savo Hospital District, Kuopio, Finland
Liisa Suominen Northern Savo Hospital District, Kuopio, Finland
Päivi Mäntylä Northern Savo Hospital District, Kuopio, Finland
Ulvi Gursoy Hospital District of Southwest Finland, Turku, Finland
Vuokko Anttonen Northern Ostrobothnia Hospital District, Oulu, Finland
Kirsi Sipilä Northern Ostrobothnia Hospital District, Oulu, Finland
Sarah Pendergrass Genentech, San Francisco, CA, United States

Women's Health and Reproduction Group
Hannele Laivuori Institute for Molecular Medicine Finland, HiLIFE, University of Helsinki, Finland
Venla Kurra Pirkanmaa Hospital District, Tampere, Finland
Oskari Heikinheimo Hospital District of Helsinki and Uusimaa, Helsinki, Finland
Ilkka Kalliala Hospital District of Helsinki and Uusimaa, Helsinki, Finland
Laura Kotaniemi-Talonen Pirkanmaa Hospital District, Tampere, Finland
Kari Nieminen Pirkanmaa Hospital District, Tampere, Finland
Päivi Polo Hospital District of Southwest Finland, Turku, Finland
Kaarin Mäkikallio Hospital District of Southwest Finland, Turku, Finland
Eeva Ekholm Hospital District of Southwest Finland, Turku, Finland
Marja Vääräsmäki Northern Ostrobothnia Hospital District, Oulu, Finland
Outi Uimari Northern Ostrobothnia Hospital District, Oulu, Finland
Laure Morin-Papunen Northern Ostrobothnia Hospital District, Oulu, Finland
Marjo Tuppurainen Northern Savo Hospital District, Kuopio, Finland
Katja Kivinen Institute for Molecular Medicine Finland, HiLIFE, University of Helsinki, Finland
Elisabeth Widen Institute for Molecular Medicine Finland, HiLIFE, University of Helsinki, Finland
Taru Tukiainen Institute for Molecular Medicine Finland, HiLIFE, University of Helsinki, Finland
Mary Pat Reeve Institute for Molecular Medicine Finland, HiLIFE, University of Helsinki, Finland
Mark Daly Institute for Molecular Medicine Finland, HiLIFE, University of Helsinki, Finland
Liu Aoxing Institute for Molecular Medicine Finland, HiLIFE, University of Helsinki, Finland
Eija Laakkonen University of Jyväskylä, Jyväskylä, Finland
Niko Välimäki University of Helsinki, Helsinki, Finland
Lauri Aaltonen Hospital District of Helsinki and Uusimaa, Helsinki, Finland
Johannes Kettunen Northern Ostrobothnia Hospital District, Oulu, Finland
Mikko Arvas Finnish Red Cross Blood Service, Helsinki, Finland
Jeffrey Waring Abbvie, Chicago, IL, United States
Bridget Riley-Gillis Abbvie, Chicago, IL, United States
Mengzhen Liu Abbvie, Chicago, IL, United States
Janet Kumar GlaxoSmithKline, Brentford, United Kingdom
Kirsi Auro GlaxoSmithKline, Brentford, United Kingdom
Andrea Ganna Institute for Molecular Medicine Finland, HiLIFE, University of Helsinki, Finland
Sarah Pendergrass Genentech, San Francisco, CA, United States

FinnGen Analysis working group
Justin Wade Davis Abbvie, Chicago, IL, United States
Bridget Riley-Gillis Abbvie, Chicago, IL, United States
Danjuma Quarless Abbvie, Chicago, IL, United States
Fedik Rahimov Abbvie, Chicago, IL, United States
Sahar Esmaeeli Abbvie, Chicago, IL, United States

Slavé Petrovski Astra Zeneca, Cambridge, United Kingdom
Eleonor Wigmore Astra Zeneca, Cambridge, United Kingdom
Adele Mitchell Biogen, Cambridge, MA, United States
Benjamin Sun Biogen, Cambridge, MA, United States
Ellen Tsai Biogen, Cambridge, MA, United States
Denis Baird Biogen, Cambridge, MA, United States
Paola Bronson Biogen, Cambridge, MA, United States
Ruoyu Tian Biogen, Cambridge, MA, United States
Stephanie Loomis Biogen, Cambridge, MA, United States
Yunfeng Huang Biogen, Cambridge, MA, United States
Joseph Maranville Celgene, Summit, NJ, United States/ Bristol Myers Squibb, New York, NY, United States
Shameek Biswas Celgene, Summit, NJ, United States/ Bristol Myers Squibb, New York, NY, United States
Elmutaz Mohammed Celgene, Summit, NJ, United States/ Bristol Myers Squibb, New York, NY, United States
Samir Wadhawan Celgene, Summit, NJ, United States/ Bristol Myers Squibb, New York, NY, United States
Erika Kvikstad Celgene, Summit, NJ, United States/ Bristol Myers Squibb, New York, NY, United States
Minal Caliskan Celgene, Summit, NJ, United States/ Bristol Myers Squibb, New York, NY, United States
Diana Chang Genentech, San Francisco, CA, United States
Julie Hunkapiller Genentech, San Francisco, CA, United States
Tushar Bhangale Genentech, San Francisco, CA, United States
Natalie Bowers Genentech, San Francisco, CA, United States
Sarah Pendergrass Genentech, San Francisco, CA, United States
Kirill Shkura Merck, Kenilworth, NJ, United States
Victor Neduva Merck, Kenilworth, NJ, United States
Xing Chen Pfizer, New York, NY, United States
Åsa Hedman Pfizer, New York, NY, United States
Karen S King GlaxoSmithKline, Brentford, United Kingdom
Padhraig Gormley GlaxoSmithKline, Brentford, United Kingdom
Jimmy Liu GlaxoSmithKline, Brentford, United Kingdom
Clarence Wang Sanofi, Paris, France
Ethan Xu Sanofi, Paris, France
Franck Auge Sanofi, Paris, France
Clement Chatelain Sanofi, Paris, France
Deepak Rajpal Sanofi, Paris, France
Dongyu Liu Sanofi, Paris, France
Katherine Call Sanofi, Paris, France
Tai-He Xia Sanofi, Paris, France
Beryl Cummings Maze Therapeutics, San Francisco, CA, United States
Matt Brauer Maze Therapeutics, San Francisco, CA, United States
Huilei Xu Novartis, Basel, Switzerland
Amy Cole Novartis, Basel, Switzerland
Jonathan Chung Novartis, Basel, Switzerland

Jaison Jacob Novartis, Basel, Switzerland
Katrina de Lange Novartis, Basel, Switzerland
Jonas Zierer Novartis, Basel, Switzerland
Mitja Kurki Institute for Molecular Medicine Finland, HiLIFE, University of Helsinki, Finland / Broad Institute, Cambridge, MA, United States
Samuli Ripatti Institute for Molecular Medicine Finland, HiLIFE, University of Helsinki, Finland
Mark Daly Institute for Molecular Medicine Finland, HiLIFE, University of Helsinki, Finland
Juha Karjalainen Institute for Molecular Medicine Finland, HiLIFE, University of Helsinki, Finland / Broad Institute, Cambridge, MA, United States
Aki Havulinna Institute for Molecular Medicine Finland, HiLIFE, University of Helsinki, Finland
Juha Mehtonen Institute for Molecular Medicine Finland, HiLIFE, University of Helsinki, Finland
Priit Palta Institute for Molecular Medicine Finland, HiLIFE, University of Helsinki, Finland
Shabbeer Hassan Institute for Molecular Medicine Finland, HiLIFE, University of Helsinki, Finland
Pietro Della Briotta Parolo Institute for Molecular Medicine Finland, HiLIFE, University of Helsinki, Finland
Wei Zhou Broad Institute, Cambridge, MA, United States
Mutaamba Maasha Broad Institute, Cambridge, MA, United States
Shabbeer Hassan Institute for Molecular Medicine Finland, HiLIFE, University of Helsinki, Finland
Susanna Lemmelä Institute for Molecular Medicine Finland, HiLIFE, University of Helsinki, Finland
Manuel Rivas University of Stanford, Stanford, CA, United States
Aarno Palotie Institute for Molecular Medicine Finland, HiLIFE, University of Helsinki, Finland
Arto Lehisto Institute for Molecular Medicine Finland, HiLIFE, University of Helsinki, Finland
Andrea Ganna Institute for Molecular Medicine Finland, HiLIFE, University of Helsinki, Finland
Vincent Llorens Institute for Molecular Medicine Finland, HiLIFE, University of Helsinki, Finland
Hannele Laivuori Institute for Molecular Medicine Finland, HiLIFE, University of Helsinki, Finland
Mari E Niemi Institute for Molecular Medicine Finland, HiLIFE, University of Helsinki, Finland
Taru Tukiainen Institute for Molecular Medicine Finland, HiLIFE, University of Helsinki, Finland
Mary Pat Reeve Institute for Molecular Medicine Finland, HiLIFE, University of Helsinki, Finland
Henrike Heyne Institute for Molecular Medicine Finland, HiLIFE, University of Helsinki, Finland

Nina Mars Institute for Molecular Medicine Finland, HiLIFE, University of Helsinki, Finland
Kimmo Palin University of Helsinki, Helsinki, Finland
Javier Garcia-Tabuenca University of Tampere, Tampere, Finland
Harri Siirtola University of Tampere, Tampere, Finland
Tuomo Kiiskinen Institute for Molecular Medicine Finland, HiLIFE, University of Helsinki, Finland
Jiwoo Lee Institute for Molecular Medicine Finland, HiLIFE, University of Helsinki, Finland / Broad Institute, Cambridge, MA, United States
Kristin Tsuo Institute for Molecular Medicine Finland, HiLIFE, University of Helsinki, Finland / Broad Institute, Cambridge, MA, United States
Amanda Elliott Institute for Molecular Medicine Finland, HiLIFE, University of Helsinki, Finland / Broad Institute, Cambridge, MA, United States
Kati Kristiansson THL Biobank / The National Institute of Health and Welfare Helsinki, Finland
Mikko Arvas Finnish Red Cross Blood Service / Finnish Hematology Registry and Clinical Biobank, Helsinki, Finland
Kati Hyvärinen Finnish Red Cross Blood Service, Helsinki, Finland
Jarmo Ritari Finnish Red Cross Blood Service, Helsinki, Finland
Miika Koskinen Helsinki Biobank / Helsinki University and Hospital District of Helsinki and Uusimaa, Helsinki
Olli Carpén Helsinki Biobank / Helsinki University and Hospital District of Helsinki and Uusimaa, Helsinki
Johannes Kettunen Northern Finland Biobank Borealis / University of Oulu / Northern Ostrobothnia Hospital District, Oulu, Finland
Katri Pylkäs University of Oulu, Oulu, Finland
Marita Kalaoja University of Oulu, Oulu, Finland
Minna Karjalainen University of Oulu, Oulu, Finland
Tuomo Mantere Northern Finland Biobank Borealis / University of Oulu / Northern Ostrobothnia Hospital District, Oulu, Finland
Eeva Kangasniemi Finnish Clinical Biobank Tampere / University of Tampere / Pirkanmaa Hospital District, Tampere, Finland
Sami Heikkinen University of Eastern Finland, Kuopio, Finland
Arto Mannermaa Biobank of Eastern Finland / University of Eastern Finland / Northern Savo Hospital District, Kuopio, Finland
Eija Laakkonen University of Jyväskylä, Jyväskylä, Finland
Samuel Heron University of Turku, Turku, Finland
Dhanaprakash Jambulingam University of Turku, Turku, Finland
Venkat Subramaniam Rathinakannan University of Turku, Turku, Finland
Nina Pitkänen Auria Biobank / University of Turku / Hospital District of Southwest Finland, Turku, Finland

Biobank directors

Lila Kallio Auria Biobank / University of Turku / Hospital District of Southwest Finland, Turku, Finland
Sirpa Soini THL Biobank / The National Institute of Health and Welfare Helsinki, Finland

Jukka Partanen Finnish Red Cross Blood Service / Finnish Hematology Registry and Clinical Biobank, Helsinki, Finland
Eero Punkka Helsinki Biobank / Helsinki University and Hospital District of Helsinki and Uusimaa, Helsinki
Raisa Serpi Northern Finland Biobank Borealis / University of Oulu / Northern Ostrobothnia Hospital District, Oulu, Finland
Johanna Mäkelä Finnish Clinical Biobank Tampere / University of Tampere / Pirkanmaa Hospital District, Tampere, Finland
Veli-Matti Kosma Biobank of Eastern Finland / University of Eastern Finland / Northern Savo Hospital District, Kuopio, Finland
Teijo Kuopio Central Finland Biobank / University of Jyväskylä / Central Finland Health Care District, Jyväskylä, Finland

FinnGen Teams

Administration

Anu Jalanko Institute for Molecular Medicine Finland, HiLIFE, University of Helsinki, Finland
Huei-Yi Shen Institute for Molecular Medicine Finland, HiLIFE, University of Helsinki, Finland
Risto Kajanne Institute for Molecular Medicine Finland, HiLIFE, University of Helsinki, Finland
Mervi Aavikko Institute for Molecular Medicine Finland, HiLIFE, University of Helsinki, Finland

Analysis

Mitja Kurki Institute for Molecular Medicine Finland, HiLIFE, University of Helsinki, Finland / Broad Institute, Cambridge, MA, United States
Juha Karjalainen Institute for Molecular Medicine Finland, HiLIFE, University of Helsinki, Finland / Broad Institute, Cambridge, MA, United States
Pietro Della Briotta Parolo Institute for Molecular Medicine Finland, HiLIFE, University of Helsinki, Finland
Arto Lehisto Institute for Molecular Medicine Finland, HiLIFE, University of Helsinki, Finland
Juha Mehtonen Institute for Molecular Medicine Finland, HiLIFE, University of Helsinki, Finland
Wei Zhou Broad Institute, Cambridge, MA, United States
Masahiro Kanai Broad Institute, Cambridge, MA, United States
Mutaamba Maasha Broad Institute, Cambridge, MA, United States

Clinical Endpoint Development

Hannele Laivuori Institute for Molecular Medicine Finland, HiLIFE, University of Helsinki, Finland
Aki Havulinna Institute for Molecular Medicine Finland, HiLIFE, University of Helsinki, Finland

Susanna Lemmelä Institute for Molecular Medicine Finland, HiLIFE, University of Helsinki, Finland
Tuomo Kiiskinen Institute for Molecular Medicine Finland, HiLIFE, University of Helsinki, Finland
L. Elisa Lahtela Institute for Molecular Medicine Finland, HiLIFE, University of Helsinki, Finland
Matti Peura Institute for Molecular Medicine Finland, HiLIFE, University of Helsinki, Finland

Communication

Mari Kaunisto Institute for Molecular Medicine Finland, HiLIFE, University of Helsinki, Finland

Data Management and IT Infrastructure

Elina Kilpeläinen Institute for Molecular Medicine Finland, HiLIFE, University of Helsinki, Finland
Timo P. Sipilä Institute for Molecular Medicine Finland, HiLIFE, University of Helsinki, Finland
Georg Brein Institute for Molecular Medicine Finland, HiLIFE, University of Helsinki, Finland
Oluwaseun A. Dada Institute for Molecular Medicine Finland, HiLIFE, University of Helsinki, Finland
Awaisa Ghazal Institute for Molecular Medicine Finland, HiLIFE, University of Helsinki, Finland
Anastasia Shcherban Institute for Molecular Medicine Finland, HiLIFE, University of Helsinki, Finland

Genotyping

Kati Donner Institute for Molecular Medicine Finland, HiLIFE, University of Helsinki, Finland
Timo P. Sipilä Institute for Molecular Medicine Finland, HiLIFE, University of Helsinki, Finland

Sample Collection Coordination

Anu Loukola Helsinki Biobank / Helsinki University and Hospital District of Helsinki and Uusimaa, Helsinki

Sample Logistics

Päivi Laiho THL Biobank / The National Institute of Health and Welfare Helsinki, Finland
Tuuli Sistonen THL Biobank / The National Institute of Health and Welfare Helsinki, Finland
Essi Kaiharju THL Biobank / The National Institute of Health and Welfare Helsinki, Finland
Markku Laukkanen THL Biobank / The National Institute of Health and Welfare Helsinki, Finland

Elina Järvensivu THL Biobank / The National Institute of Health and Welfare Helsinki, Finland
Sini Lähteenmäki THL Biobank / The National Institute of Health and Welfare Helsinki, Finland
Lotta Männikkö THL Biobank / The National Institute of Health and Welfare Helsinki, Finland
Regis Wong THL Biobank / The National Institute of Health and Welfare Helsinki, Finland

Registry Data Operations

Hannele Mattsson THL Biobank / The National Institute of Health and Welfare Helsinki, Finland
Kati Kristiansson THL Biobank / The National Institute of Health and Welfare Helsinki, Finland
Susanna Lemmelä Institute for Molecular Medicine Finland, HiLIFE, University of Helsinki, Finland
Sami Koskelainen THL Biobank / The National Institute of Health and Welfare Helsinki, Finland
Tero Hiekkalinna THL Biobank / The National Institute of Health and Welfare Helsinki, Finland
Teemu Paajanen THL Biobank / The National Institute of Health and Welfare Helsinki, Finland

Sequencing Informatics

Priit Palta Institute for Molecular Medicine Finland, HiLIFE, University of Helsinki, Finland
Kalle Pärn Institute for Molecular Medicine Finland, HiLIFE, University of Helsinki, Finland
Shuang Luo Institute for Molecular Medicine Finland, HiLIFE, University of Helsinki, Finland
Vishal Sinha Institute for Molecular Medicine Finland, HiLIFE, University of Helsinki, Finland

Trajectory Team

Tarja Laitinen Pirkanmaa Hospital District, Tampere, Finland
Harri Siirtola University of Tampere, Tampere, Finland
Javier Gracia-Tabuenca University of Tampere, Tampere, Finland
Mika Helminen University of Tampere, Tampere, Finland
Tiina Luukkaala University of Tampere, Tampere, Finland
Iida Vähätalo University of Tampere, Tampere, Finland

Data protection officer

Tero Jyrhämä Institute for Molecular Medicine Finland, HiLIFE, University of Helsinki, Finland

FinBB - Finnish biobank cooperative

Marco Hautalahti
Laura Mustaniemi
Mirkka Koivusalo
Sarah Smith
Tom Southerington
